# Personal Traits Underlying Environmental Preferences: A Discrete Choice Experiment

**DOI:** 10.1371/journal.pone.0089603

**Published:** 2014-02-20

**Authors:** Mario Soliño, Begoña A. Farizo

**Affiliations:** 1 National Institute for Agriculture and Food Research and Technology (INIA), Forest Research Centre (CIFOR), Madrid, Spain; 2 Sustainable Forest Management Research Institute, University of Valladolid & INIA, Palencia, Spain; 3 Institute for Public Goods and Policies (IPP), Spanish National Research Council (CSIC), Madrid, Spain; George Mason University, United States of America

## Abstract

Personality plays a role in human behavior, and thus can influence consumer decisions on environmental goods and services. This paper analyses the influence of the big five personality dimensions (extraversion, agreeableness, conscientiousness, neuroticism and openness) in a discrete choice experiment dealing with preferences for the development of an environmental program for forest management in Spain. For this purpose, a reduced version of the Big Five Inventory survey (the BFI-10) is implemented. Results show a positive effect of openness and extraversion and a negative effect of agreeableness and neuroticism in consumers' preferences for this environmental program. Moreover, results from a latent class model show that personal traits help to explain preference heterogeneity.

## Introduction

It is broadly recognized that the specific behavior of individuals is conditioned by individual factors, home-site factors [1], [2], [3], [4], [5] and a set of formal rules and socially accepted informal rules [6], such as those of family or culture. Personality also plays a role in human behavior and thus, can influence environmental concerns [7], environmental engagement [8] and consumer decisions on environmental goods and services [9].

Several environmental studies have considered the influence of attitudes or tastes [10], [11], [12], [13] and psychological constructs [14], [15] on individual preferences. The influence of personality dimensions on environmental preferences has been less examined. To correct this gap, a reduced version of the BFI such as the BFI-10 is a suitable instrument to incorporate personality traits in environmental valuation, since it is a recommended approach for research in which participant time is limited and when personality assessment would otherwise be impossible. Environmental economics and more specifically, environmental valuation, uses survey-based instruments to analyze consumer behavior among natural and environmental resources. In this case, the BFI-10 shows acceptable psychometric properties [16] and is a suitable instrument to introduce the personality traits into the economic analysis.

The Big Five Inventory (BFI) is a personality survey based on a set of phrases used to measure the Big Five dimensions of personality, i.e., extraversion, agreeableness, conscientiousness, neuroticism and openness [17]. Although BFI can be answered in less than 10 minutes, there is a growing demand for shorter instruments [18], [19], [20]. Reduced scales of BFI cannot be used as a common approach to assess personality [16], [20], but is a good alternative to implement personality in other scientific disciplines different from psychology, such as economics.

The paper is structured as follows. Section 2 presents the theoretical underpinnings of Discrete Choice Experiments and the BFI-10. Section 3 outlines the questionnaire used to analyze the influence of personality traits on environmental valuation. In Section 4 we present the main results of the study. Finally, Section 5 is devoted to conclusions and discussion.

## Materials and Methods

### The BFI-10

The Big Five Traits allow us to classify people by analyzing their ratings in a set of short phrases characterizing five independent personality dimensions. Following [16], the BFI-10 was implemented in order to analyze the influence of personality traits in environmental valuation. Ten short phrases were used to represent the big five personality dimensions, with just 2 items per dimension (extraversion, agreeableness, conscientiousness, neuroticism and openness). Individuals rate these 10 short phrases (Table 1) describing their personality on a five-step scale from 1 (disagree strongly) to 5 (agree strongly).

**Table 1:** Short phrase items used in the BFI-10.

Dimension	Statements describing personality
Extraversion	Is reserved(−)
	Is outgoing, sociable
Agreeableness	Is generally trusting
	Tends to find fault with others(−)
Conscientiousness	Tends to be lazy(−)
	Does a thorough job
Neuroticism	Is relaxed, handles stress well(−)
	Gets nervous easily
Openness	Has few artistic interests(−)
	Has an active imagination

(−) Reversed-scored item.

A comprehensive analysis of different interpretations of these dimensions is presented in [21], [22]. For the purpose of this article, we summarize the focal interpretation of the five personality dimensions:

- Extraversion: people classified as “extroverts” show social adaptability and interpersonal involvement, and they are talkative, assertive, active, outgoing, and outspoken. Therefore, we expect a positive likelihood of these people contributing to the development of a new environmental program.
- Agreeableness: this dimension implies pro-social behavior, and people scoring high on agreeableness tend to be sympathetic, kindly, appreciative, affectionate, soft-hearted, warm and generous. There is no prior expectation for the influence of this dimension on choices for the environmental program. Nevertheless, a significant positive effect could be interpreted as a warm-glow bias (friendly behavior).
- Conscientiousness: they are organized, thorough, playful, efficient, responsible and reliable. They are “global-thinkers” and, due to the regional character of the proposed environmental program, we expect a negative or non-significant relationship between high scores in this dimension and the probability of accepting a small (not far-reaching) environmental program.
- Neuroticism: typically, people classified as “neurotics” are tense, anxious, nervous, moody, and tend to worry. Therefore, a negative relationship is expected between high scores in this dimension and the probability of accepting a novel environmental program. These personalities might tend to choose the status quo option rather an option of change [9]. Moreover, we expect extreme behaviors and a high dispersion of responses from neurotics.
- Openness: these are people open to experience; they have wide interests and are imaginative, intelligent and original. Therefore, we expect a positive likelihood of these people contributing to the development of a new environmental program.

### Discrete choice experiments

Discrete Choice Experiments (DCEs) simulate markets in which different environmental goods and services compete in a realistic trade-off manner. Individuals choose between different alternatives involving environmental attributes, according to their own preferences and budget constraints. DCEs allow researchers to infer consumers' preferences and implicit prices for several characteristics embedded in a prospective policy or environmental program. Since accounting for unobserved heterogeneity is a matter of importance in the estimation of behavioral models, two mixed approaches are applied: a Random Parameter Logit (RPL) model [23] and a Latent Class (LC) Model [24].

The RPL model introduces unobserved preference heterogeneity, i.e., allows the coefficients of observed variables to vary randomly among people rather than being fixed. In RPL models, the individual's *i* indirect utility function (*V_i_*) is usually represented as a linear additive expression:
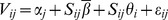
(1)where *α_j_* is an alternative specific constant (ASC) for each option (*j* =  1,2,...,J) in the choice set, *S_ij_* is the associated attribute vector, 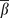 is the vector of population mean preference values, *θ_i_* represents the deviations in individual preferences with respect to the mean values, and 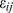 is an *i.i.d.* type I extreme value random component of utility which cannot be observed by the researcher. Coefficients *β* vary in the population with density ƒ(β|Ω), with Ω denoting the parameters of density. If we assume that the individual's preferences, as represented by *β_i_*  =  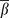 +*θ_i_*, follow the same decision heuristics for all choice (*t =  1,2,..,T*), the probability of individual *i*'s observed sequence of choices [*y_1_, y_2_,...,y_T_*] is calculated by solving the integral:
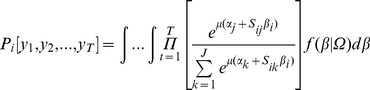
(2)where j is the alternative chosen in choice occasion t and μ is a scale parameter.

As pointed above, another way to capture taste variations is through LC or by a combination of LC and RPL. In this study we apply a latent class model with common and specific random parameters. Latent class has been long applied in environmental modeling, but not many studies combine both mixed approaches. Some examples of the dynamism of LC can be seen in [25], describing the choice of excursions in natural protected areas; [26] for transport issues; or [4], [27] for valuing environmental goods.

Following [24], the model has several classes (*x*) for the probability that case *i* selects alternative *j* at the replication *t,* given attribute value 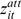 (characteristics of alternatives), covariates 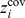, used to predict class membership (*x*), and random parameters (RP) denoted by *F_di_* being *d* the number of factors. The *F_di_* are assumed to be standard normally distributed and mutually independent (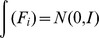) where *I* is the identity matrix.

In a general case, with a conditional logit model, the response probability is:
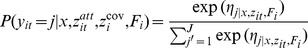
(3)where 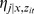 is the systematic component in the utility of alternative *j* at replication *t* given that case *i* belongs to latent class *x*. The linear model is:



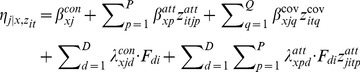
(4)The term 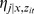 is a linear function of an alternative specific constant 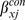, attribute effects 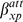 and random effects. The first term (
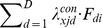
) defines random effects for the alternative specific constants, and the following terms define random effects for the attributes.

In a latent class choice model with random parameters, the probability density has this form:
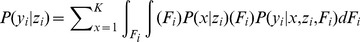
(5)where



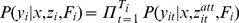
(6)A multinomial logit is specified in which class membership is regressed on covariates;
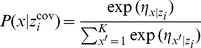
(7)with linear term 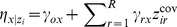, being 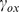 the constant corresponding to latent class *x* and 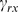 the effect of the *r*th covariate for the Class *x*.

In our study, personal traits are included as covariates to explain belonging to a specific class, assuming that members of the same class share a pattern of choice. On the other hand, we assume that attributes from the discrete choice experiment behave randomly. This specification allows us to identify whether one or more attributes are not random to a specific class, improving the accuracy of the model.

### The environmental valuation questionnaire

The choice experiment aims to quantify the population's preferences for several externalities associated with a program developing resin-tapping in Spain, i.e., biodiversity of flora, employment generated by the strengthening of the sector, reduced risk of fire, the presence in the market of “certified products” that incorporate natural resin, and the duration and the annual cost of the program. The levels presented are detailed in Table 2.

**Table 2:** Attributes and Levels of the Choice Experiment.

Attribute	Levels	Variable
Biodiversity of flora	Low*	*BioL*
	Medium	*BioM*
	High	*BioH*
Employment (number of new jobs)	0*, 50, 100	*Employ*
Risk of forest fires	High*	*FireH*
	Medium	*FireM*
	Low	*FireL*
Presence of certified products that incorporate natural resin	Low*	*CertL*
	Medium	*CertM*
	High	*CertH*
Duration (in years)	0*, 5, 10, 15	*Time*
Cost (€/year via increase of taxes)	0*, 6, 12, 18	*Tax*

* Status Quo level.

The discrete choice experiment is implemented in an environmental valuation questionnaire structured in 4 sections. Section 1 contains questions relating to the relationships between the interviewee and forests. The second section examines knowledge about resin-tapping activities. Section 3 contains the discrete choice experiment. The choice sets were designed following an optimal in difference design as proposed by [28]. From an optimal design size of 18 choice cards, a blocking strategy was performed and each respondent was confronted with nine choice cards containing two prospective programs and the status quo scenario (where the program is not implemented and the individual does not pay any extra fee). The experimental design is balanced, i.e. all the attribute levels are shown the same number of times to each individual. In general, unbalanced designs should be avoided where possible because statistical power differs within attribute levels and/or between attributes, and artificial correlations with grand means or model intercepts are introduced [29].

Finally, the fourth section is focused on socio-economic characteristics and includes the BFI-10 questions. The questionnaire was administered in September and October 2012 to 2,224 individuals. The target population was adult dwellers from Castilla y León, since resin-tapping activities in Spain are concentrated in this region (located in central Spain). The database used for this analysis is available on request from the authors.

## Results

From the individuals' ratings on the BFI-10 short-phrases items, the average score is calculated for each of the big five personality dimensions. The results are shown in Table 3. Conscientiousness shows the higher value and neuroticism shows the lower one, whereas extraversion, agreeableness and openness show similar values. Low correlations are detected for the five dimensions of personality. This is a good indicator of the independence of these personality dimensions.

**Table 3:** Average score and correlations for the big five personality dimensions.

Personality dimension	Score*		Matrix of correlations**				
	Mean	Std. Dev.	Extrav.	Agreeab.	Conscient.	Neurot.	Open.
Extraversion	3.445	0.945	1.000				
Agreeableness	3.443	0.798	0.141	1.000			
Conscientiousness	4.085	0.793	0.173	0.170	1.000		
Neuroticism	2.781	0.960	−0.092	−0.155	−0.072	1.000	
Openness	3.501	0.881	0.177	0.061	0.195	−0.080	1.000

* Range from 1 to 5.

** All correlations are significant at the 0.01 level (2-tailed).

Table 4 presents the results of the RPL estimations using 500 Halton draws (Train, 2003) and NLOGIT 5.0 software. All attributes, apart from the cost and the Alternative Specific Constant (ASC) were assumed to be normally distributed. Differences of the Akaike Information Criterion (AIC) between a baseline model (without including personality traits) and the expanded-BFI model favored the selection of the last one. The purpose of using these explanatory variables in our analysis is focused on getting a better understanding on the relation between personality traits and choices, to help us explaining why individuals prefer certain things over others. The ASC was introduced in the models as a dummy variable taking value 1 to indicate that the individual does not choose the status quo scenario. The estimated coefficient for the ASC is statistically non-significant. This result suggests that individual heterogeneity is correctly captured by the model.

**Table 4:** Random Parameters Model results.

Variable	Baseline model		Expanded-BFI model	
	Coef. (Std. Err.)	Std. Dev. of parameter distributions (Std. Err.)	Coef. (Std. Err.)	Std. Dev. of parameter distributions (Std. Err.)
*Employ*	0.025* (0.001)	0.031* (0.001)	0.025* (0.001)	0.031* (0.001)
*Time*	0.023* (0.008)	0.248* (0.008)	0.025* (0.008)	0.239* (0.008)
*BioM*	0.145* (0.027)	0.381* (0.048)	0.160* (0.027)	0.372* (0.055)
*BioH*	0.231* (0.026)	0.474* (0.042)	0.240* (0.026)	0.494* (0.041)
*FireM*	0.340* (0.025)	0.326* (0.055)	0.353* (0.026)	0.405* (0.050)
*FireL*	1.070* (0.039)	1.115* (0.043)	1.064* (0.039)	1.107* (0.044)
*CertM*	0.168* (0.024)	0.308* (0.050)	0.174* (0.025)	0.341* (0.045)
*CertH*	0.337* (0.026)	0.505* (0.041)	0.335* (0.027)	0.511* (0.041)
*ASC*	0.772* (0.085)	fixed	−0.036 (0.479)	fixed
*Tax*	−0.067* (0.004)	fixed	−0.067* (0.004)	fixed
*Extraversion*	-		0.205* (0.063)	
*Agreeableness*	-		−0.260* (0.075)	
*Conscientiousness*	-		−0.121 (0.074)	
*Neuroticism*	-		−0.132** (0.062)	
*Openness*	-		0.525* (0.067)	
Log likelihood	−12,813.400		−12,760.600	
AIC	25,662.80		25,580.01	
Pseudo-R^2^ (adj.)	0.4170		0.4194	
#. indiv.	2,224			
# observ.	20,016			

* p<0.01 ** p<0.05.

From these results (Table 4) we can state that personality dimensions have an effect on the choices individuals make. Our results show that the most influential trait is *openness*, that is, individuals showing a high score in *openness* (people open to new experiences) are more prone to choose the options for protection than people with other dominant traits. All covariates have the expected sign except *agreeableness*, for which we did not have any a priori expectations about the sign. Since stated preference techniques are prone to get a positive answer from individuals whatever they are offered, our hypothesis was that individuals who show this trait could favor the yea-saying bias.

It would thus be worthwhile to know which aspects of the personality make us value certain attributes over others; that is, the influence of personality traits on preferences for environmental programs. For this we run a latent class model using Latent Gold 4.5 software. In order to benefit from the virtues of the RPL models, we consider that some attributes may have just one source of variability (for *FireL*, *FireM*, *Time*, *CertM* and *CertH*) while for others (*Employ*, *BioM*, *BioH* and *Cost*) we identified two, one dependent on the class and another constant for all the classes. In other words, we assume that for some attributes dispersion around the mean estimates is the same for all individuals, like the RPL shown above, while for some other attributes we also have identified variability in the class (intra-class).

Table 5 shows the mixed LC-RPL model, where the *SDPD* column contains the Standard Deviations of the estimates for the classes and the last column, *Common SDPD* shows the Standard Deviations for the variables, regardless of the class. This decomposition of variability has the advantage of informing us about possible sources of heterogeneity. For example, the estimates for medium risk of fire (*FireM*) are positive for all classes and show high variability for everybody, while for low risk (*FireL*) the variation is due to the composition of the classes. For some, this is something desirable, while for others it seems it is enough to achieve the medium level; some risk of fire might be acceptable for certain groups of the society because low risk could not be credible or because the cost of achieving that goal may be disproportionate. Fire variables were expected to behave quite homogeneously and have low variability. On the contrary, *BioM* and *BioH* show great inter- and intra-class variability, as shown by the common and by-class SD. *Cost* is another variable with great variation. Traditionally, the cost of the program parameter is considered as fixed, since the elicitation of willingness to pay (WTP) measures complicates the computation. In this study we are not interested in getting the benefits (in terms of willingness to pay) of the program but to control and explain heterogeneity of preferences, thus *Cost* is considered to have a random nature. Again, two sources of variability have been identified, one common to all individuals no matter the class, and another for the classes. As with the *BioM* and *BioH*, for some classes the decisions on this attribute are not random, as is the case for classes 3 and 4. If we go into the composition of the classes attending to their personality traits, we can see that class 4 is the one with lower random behavior and *Openness* is the trait of their members (around 13% of the sample). The most valued aspect of the program for this class is biodiversity followed by fires, both a medium level but valuing positively high levels of biodiversity and negatively low levels of risk of fires.

**Table 5:** Latent Class Model results.

Variables	Class 1		Class 2		Class 3		Class 4		Class 5		Class 6		Class 1–6
	Coef.	SDPD	Coef.	SDPD	Coef.	SDPD	Coef.	SDPD	Coef.	SDPD	Coef.	SDPD	Common SDPD
*Employ*	0.019*	0.009*	0.226*	0.230*	−0.084*	−0.131*	0.085*	−0.012	0.019*	−4.124*	0.120*	0.124*	0.007*
*Time*	0.037*	−0.018***	0.457*	−0.463*	−0.911*	−0.832*	0.539*	0.047	0.796*	0.136*	0.587*	1.403*	n.s.
*BioM*	0.166*	0.080***	−3.739*	−1.931*	4.279	1.979	2.934*	0.218	−0.524	0.160	3.248*	−1.437*	0.179*
*BioH*	0.258*	0.180*	3.025*	1.576*	−6.657***	−2.455	1.233***	−0.908	−1.352*	0.020	−0.964	7.663*	−0.226*
*FireM*	0.324*	n.s.	1.152*	n.s.	0.373	n.s.	2.304*	n.s.	1.954*	n.s.	4.835*	n.s.	0.335*
*FireL*	1.098*	0.743*	1.233*	0.675*	−0.157	0.166	1.061*	0.287	7.326*	4.478*	2.285*	−2.794*	n.s.
*CertM*	0.243*	0.141*	5.152*	3.304*	1.725***	1.175***	−1.114	0.296	1.048*	−1.831*	−0.160	−5.857*	n.s.
*CertH*	0.428*	0.170*	−0.273***	−0.325*	4.896*	3.382*	0.329	0.779*	−0.150	−4.960*	−1.807*	−0.813	n.s.
*ASC*	1.024*	Fixed	−0.151	fixed	1.102*	fixed	0.336	fixed	−1.009***	fixed	−1.301	fixed	fixed
*Tax*	−0.021*	−0.050*	−0.265*	−0.090*	−1.116*	−0.035	−0.122***	−0.068	0.103*	0.439*	−1.110*	−0.698*	0.072*
*Extraversion*	0.004		−0.146*		0.011		0.090		0.203*		−0.162		
*Agreeableness*	−0.137*		0.255*		0.001		−0.160		−0.112		0.153		
*Conscientiousness*	−0.040		0.165*		0.061		−0.128		−0.069		0.010		
*Neuroticism*	0.021		−0.086		0.120**		−0.149		0.004		0.089		
*Openness*	0.184*		−0.039		−0.492*		0.244*		0.222*		−0.118		
pseudo R^2^	44.5		85.83		96.82		71.76		88.19		85.97		75.57
Class Size (%)	37.76		21.81		13.92		12.96		10.56		2.99		100.00

* p<0.01 ** p<0.05 *** p<0.10.

SDPD refers to standard deviation of parameter distributions.

Classes 1, 2 and 6 are at the other end, showing high variation of their mean parameters. Attending to the personality traits, class 6, which only represents 3% of the sample, has none that could describe their mean personality. Class 2, with almost 22% of the sample, groups the people with low *extraversion* and positive and high scores on *agreeableness* and *conscientiousness*. Class 1 is the biggest with almost 38% of the participants. They have high scores on *openness* (positive) and on *agreeableness* (negative). For people in this group cost is the least important attribute and *FireL* the most important, and they will choose any improvement for the management of these areas. As expected, this group has a high variability on all the parameters. This may be due, among other things, to the fact that is the most populated class.

A trait that deserves attention is *neuroticism,* which is significant only for class 3 (14% of the sample) together with a negative *openness*. Our results confirm [30], indicating that neuroticism could be a weaker predictor of a range of outcomes. As expected, few aspects of the environmental program are appreciated by this group. Key attributes like fire are not even significant. Class 5 (around 11% of the sample) is pro-environmental and the positive sign of cost could have an explanation in the positive and significant traits for this class: *extraversion* and *openness*.

Finally, in Section 2 we hypothesized that a positive and significant Agreeableness could lead to a warm glow behavior, but our results shows that the assertive aspect of Extraversion, combined with other traits could be more prone to this kind of bias (see Class 5). Our experiment is not big enough to provide evidence or establish these relations; thus only further research could lead us to find if those relations are possible.

## Conclusions

This paper relates personality traits directly with environmental preferences. It shows that personality plays a role in human behavior and thus, it can influence consumer decisions on environmental goods and services. In environmental valuation with stated preference methods a hypothetical market is simulated through a survey instrument. In order to avoid fatigue effects, the survey time is very limited, thus a key issue for implementing personality traits in environmental economics is the time required to answer the psychological questions. For this reason, a reduced version of Big Five Inventory with acceptable psychometric properties (the BFI-10) was applied in order to analyze the influence of personality traits in a discrete choice experiment. Therefore, this paper uses a psychometric approach to highlight the relevance of personality traits in consumer decisions, relating directly personality to economic behavior. Other approaches such as the New Ecological Paradigm scale [31] have connected pro-environmental behavior with economic behavior (i.e. a higher willingness to pay). Moreover, in the literature are numerous examples connecting different types of behavior and, what it is very interesting too, connecting the “values” such as the Schwartz Value Theory [32], [33] to explain individual preferences [34]. Discussions on these issues can be found in several papers from the 2011 International Choice Modelling Conference [35]. We have tried with this experiment to go a step backwards, since we agree in that values are partly inspired by personality. But we recognize that this study is a short piece of the research on the influence of personality on individual preferences, and other relations will be tested in the future.

This study brings new insights on the relationship between personality and environmental values. Results show a positive effect of openness and extraversion and a negative effect of agreeableness and neuroticism in consumers' preferences for this environmental program. Nevertheless, these effects are diverse and affect the heterogeneity of preferences, the preferred characteristics and the desirability of the program itself. Further research is necessary to explain, for example, why estimates from individuals with high scores on conscientiousness have higher random behavior than those showing a neuroticism trait. That is, to better explain the relationship between personality traits and tastes and preferences. In summary, this work constitutes a new step to understanding the involvement of individuals in environmental conservation and could help to better design appropriate ways to reach certain groups and ensure the success of environmental and social goals.
